# Supplementary material for: Post-Discharge non-invasive ventilation for hypercapnic respiratory failure: Outcomes in a Rural Cohort
Source: PLoS One. 2025 Apr 16;20(4):e0321420. doi: 10.1371/journal.pone.0321420 (PMC12002495; doi:10.1371/journal.pone.0321420)
Supplement: Attachment — Submitted filename: Reviewers comments for NIV study_Feb8.docx [file pone.0321420.s002.docx]

Response to reviewers comments for NIV study

Editorial comments

Please remove any funding-related text from the manuscript and let us know how you would like to update your Funding Statement. Currently, your Funding Statement reads as follows: “The authors received no specific funding for this work.”.
Please include your amended statements within your cover letter; we will change the online submission form on your behalf.

**Funding related text removed from the manuscript as advised.**

**Please add the following funding information on the submission form:**

**“Research reported in this publication was supported by the National Institute Of General Medical Sciences of the National Institutes of Health under Award Number 5U54GM104942-08. The content is solely the responsibility of the authors and does not necessarily represent the official views of the National Institutes of Health.”**

**Thank you.**

Please provide an amended Competing Interests Statement that explicitly states this commercial funder, along with any other relevant declarations relating to employment, consultancy, patents, products in development, marketed products, etc.

Please include your amended Competing Interests Statement within your cover letter. We will change the online submission form on your behalf.

**Amended competing interests statement added to the manuscript and added the sentence “This does not alter our adherence to PLOS ONE policies on sharing data and materials.”**

Please review your reference list to ensure that it is complete and correct.

**Reference list is complete and correct.**

5. Review Comments to the Author

Please use the space provided to explain your answers to the questions above. You may also include additional comments for the author, including concerns about dual publication, research ethics, or publication ethics. (Please upload your review as an attachment if it exceeds 20,000 characters)

Reviewer #1: I would have liked more explanation on the randomization process- since the control group consisted of participants who "did not qualify for non-invasive ventilation at discharge," but the study group did not include that subset of patients then doesn't that naturally skew your results? Are the patients first randomized into control vs study groups and then determined if they met criteria for NIV at discharge? That way, there would naturally be patients in both control and study groups that did not end up meeting criteria for NIV at discharge and their data removed for purposes of this study.

**We appreciate the reviewer’s observation. Most patients in the control group were admitted by the primary team for acute-on-chronic hypercapnic respiratory failure and were not evaluated by the hospital sleep medicine service. Additionally, we included patients who were consulted but were unable to be discharged on NIV. This approach was appropriate, as the study aimed to assess whether patients who were not discharged on therapy were at higher risk for poor outcomes. As noted in Table 1, there were no significant differences between the intervention and control groups. Since this is a retrospective study based on a prospective registry, the control group was not initially randomized. To clarify, we have updated the methodology as follows:**

**“The control group consisted of hospitalized patients who had hypercapnia (PaCO2 ≥ 45 mmHg) on arterial blood gas or a VPaCO2 ≥ 51 mmHg from their venous blood gas, comorbidity of COPD or a BMI ≥ 30 Kg/m2 who were admitted during the same time period but the primary admitting team did not consult hospital sleep medicine service, and those who did not qualify for non-invasive ventilation at discharge or experienced insurance denial.”**

I would also have liked a breakdown for chief complaint causing the 12 month mortality and hospital readmissions out of curiosity to see what percentage were pulmonary related.

**Unfortunately, we could not determine specific causes of mortality as records from outside hospitals were not available. This has been listed in the limitations section. Prospective studies in on further delineation of causes for mortality are recommended.**

Reviewer #2: The article is technically sound but has notable minor methodological limitations.

Patients with a prior diagnosis of acute-on-chronic hypercapnic respiratory failure who were already on home NIV were excluded. This exclusion may bias the study population toward newly diagnosed or less severe cases, potentially limiting the applicability of the findings to patients with long-standing NIV use or more advanced disease.

We thank the reviewer for their excellent comments.

**We believe it was important to exclude patient already on NIV as the duration of use and hours of compliance could not be controlled. We agree with the reviewer that enrolling NIV naïve patients may have biased the cohort towards less severe cases. However our data shows that NIV was beneficial even in relatively less severe patients and supports the conclusion that more severe patients may also benefit.**

The retrospective data, relied on data extracted from electronic medical records and a sleep medicine registry and hence prone to selection bias, missing data, and inaccuracies in documentation. Adherence data obtained from durable medical equipment companies, may not capture the full picture of patient usage patterns and may not account for patient-reported barriers (e.g., discomfort, mask fit issues) or technical issues with the devices. Elaborating on the methods these equipment companies use to collect adherence data may provide helpful context for readers.

**We again thank the reviewer for their comments and agree with the observations. We have addressed this in the limitations section. Downloadable objective adherence data from the DME company was obtained and reviewed by the investigators and was not based solely on DME companies observations or documentation. Information on patient reported barriers was obtained from clinic notes and encounters. This has been added to the methodology section.**

Lastly, the study included patients admitted between January 2020 and July 2023, i.e. during the COVID-19 pandemic which most likely may have influenced hospitalization patterns, access to care, and outcomes, how were these factors addressed and adjusted for in the study?

**We agree with the reviewer that the COVID-19 pandemic affected all aspects of medical care, and the impact of these confounders is difficult to quantify. However, regarding acute-on-chronic hypercapnic respiratory failure, we did not observe a decrease in cases but rather a slight increase, likely due to the temporary closure of ambulatory clinics. Both the intervention and control groups were exposed to similar environmental influences. We have acknowledged the unknown impact of COVID-19 in the study’s limitations section.**

While revising your submission, please upload your figure files to the Preflight Analysis and Conversion Engine (PACE) digital diagnostic tool, [https://pacev2.apexcovantage.com/](https://nam04.safelinks.protection.outlook.com/?url=https%3A%2F%2Ftrack.editorialmanager.com%2FCL0%2Fhttps%3A%252F%252Fpacev2.apexcovantage.com%252F%2F1%2F010f0194d345c636-ee8d8209-7e4e-4606-a5b6-bef06e7c0aac-000000%2FjX-f0iDdT32edXIH3VyP1sIiMW4pupZjNDW37Mbl-8A%3D196&data=05%7C02%7Csunil.sharma%40hsc.wvu.edu%7C00a6d98ffb504b79ccd408dd4572cd9e%7Ca2d1f95f851044248ae15c596bdbd578%7C0%7C0%7C638743081862262298%7CUnknown%7CTWFpbGZsb3d8eyJFbXB0eU1hcGkiOnRydWUsIlYiOiIwLjAuMDAwMCIsIlAiOiJXaW4zMiIsIkFOIjoiTWFpbCIsIldUIjoyfQ%3D%3D%7C0%7C%7C%7C&sdata=93s6PcP9IffPFYI%2B4Z%2BI79cdcshLrW6QWqe3Ho%2FFSIM%3D&reserved=0). PACE helps ensure that figures meet PLOS requirements. To use PACE, you must first register as a user. Registration is free. Then, login and navigate to the UPLOAD tab, where you will find detailed instructions on how to use the tool. If you encounter any issues or have any questions when using PACE, please email PLOS at [figures@plos.org](mailto:figures@plos.org). Please note that Supporting Information files do not need this step.

**Done**
